# From Transparent to Opaque: A Route towards Multifunctional Parts Injected with a Single Material

**DOI:** 10.3390/ma16186219

**Published:** 2023-09-15

**Authors:** Luís D. Pedroso, António J. Pontes, António Alves, Fernando M. Duarte, Olga S. Carneiro

**Affiliations:** 1 Department of Polymer Engineering, Institute for Polymers and Composites (IPC), University of Minho, 4800-058 Guimarães, Portugal; luis_pedroso_09@hotmail.com (L.D.P.); pontes@dep.uminho.pt (A.J.P.); fduarte@dep.uminho.pt (F.M.D.); 2 Cabopol—Polymer Compounds, 2480-049 Leiria, Portugal; antonio.alves@cabopol.com

**Keywords:** graded property, transparency, haze, injection molding, polypropylene, circular economy, sustainability, recycling

## Abstract

The technological, social and economic development observed in recent decades brought an exponential increase in consumption and inherent new challenges. Recycling is one of the best solutions to minimize the environmental impact of raw materials. However, multi-material components are difficult or even impossible to recycle. The present work focuses on the reduction in the number of different materials used in multifunctional components. In particular, it intends to assess the potential of injecting molding grades of polypropylene (PP) to produce parts with transparency (haze) gradients. Firstly, several polypropylene grades of different types were identified and injected under various thermal processing conditions, i.e., injection temperature and mold temperature, in order to vary the cooling rate, influencing the growth rate of the spherulites and eventually the presence/absence of α and β crystalline zones. The injected parts’ optical properties were then characterized, and the most promising PP grades were identified and selected for subsequent work, namely grade DR 7037.01, showing the widest range of haze (from 29.2 to 68.7%). and PP070G2M, presenting the highest haze value (75.3%). Finally, in an attempt to understand the origin of the haze variations observed, the parts injected with the selected PP grades were further characterized through differential scanning calorimetry (DSC) and polarized light microscopy. It was concluded that the main factor causing the observed haze difference was, apart from the size of the spherulites, the presence of internal layers with different birefringence and, therefore, different refractive indices.

## 1. Introduction

Plastics have changed our way of living and quality of life since the second half of the 20th century. Presently, the annual production of plastic products is of nearly 400 million tons per year, and Plastic Atlas predicts that by 2025 it will reach 600 million tons, which corresponds to an increase of more than 50% of the current production [1,2]. The unrestrained use of this type of materials, and its consequent increase as post-consumption material in the Earth’s geological space, has led several scientists to classify our time as the plastic era [3]. Looking around, it is possible to find plastics in products/services of all application areas, from agriculture to aeronautics, packaging, sports, automotive industry, healthcare, civil construction, among many others [1,2,4]. The huge success of plastics can be attributed to two of their major characteristics: diversity and versatility [5]. In fact, there are thousands of polymeric materials, if the several existing grades and variants are considered, able to satisfy the most wide-ranging demands [6]. Their versatility comes not only from their easy processing, but also from their ability to be easily transformed into complex geometries [4,7,8,9,10]. Additionally, and as a general rule, when compared to other materials, plastics are cheaper, lighter, have a wide range of mechanical properties, and very good chemical resistance and thermal and electrical insulation properties [4,7,10].

The success of plastics has led to some severe problems. In fact, the colossal increase in their use in various areas of consumption and industry led to an unprecedented human development in the history of the planet [11]. However, this also led to an exponential increase in environmental pollution, with the main impact being on waterways and oceans, destroying habitats and endangering hundreds of marine species [1,11,12,13]. Additionally, the lack of economic resources and infrastructure in less developed countries led to the accumulation of these materials in inappropriate areas, which due to human or natural action are often involuntarily directed to waterways or are voluntarily directed to landfills and incineration [2]. None of these options is the most convenient, since materials should be reused or recycled at the end of the product’s lifetime. The recycling figures in the European Union are quite encouraging [2,11], but are not similar across all countries. Recycling is not always possible when multi-material products such as multilayer co-extruded films or multi-material injection molded components are considered. In these cases, the materials’ separation at the end of life and subsequent recycling, required for a circular economy, is much more difficult. Energy recovery is a popular method in these cases [14].

This paper investigates the feasibility of using a single material to produce injection-molded parts with a transparency gradient, thereby progressing towards mono-material products with property gradient. This aims to improve the end of life of these products, facilitating their recycling and, as a result, contributing to a circular economy.

There are several ways to modify the transparency of a plastic part, since light can be blocked from crossing the plastic and/or be scattered from its usual path via obstacles with dimensions greater than the wavelength of visible light [15,16,17,18]. These obstacles absorb the incident light and/or scatter it. Fillers, pigments, voids, or crystalline zones of semi-crystalline thermoplastics, among others, are examples of this kind of obstacles. Studies on the effect of thermoplastic crystallinity on optical properties revealed that the dimensions of the spherulites affect light transmission through the polymer. Transmission is higher in materials with very small spherulites, while haze is higher in parts with larger spherulites [15,17,18,19,20,21]. The crystalline structure can be found in two most common configurations: alpha and beta. The first one exists in large quantities in the polymer, whereas the second exists in very small quantities (sometimes not at all), unless the raw material is processed under the ideal conditions leading to its formation or specific nucleating agents are incorporated for this sake [15,22,23,24]. In order to vary the dimensions of the spherulites, nucleating and clarifying agents can be incorporated into the polymer matrix, which promote an increase in the number of crystallization active centers. Additives can also be added to speed up the crystallization process, favoring the appearance of alpha spherulites, which will be smaller and have less influence on the optical properties of the final part [19]. As a general rule, the lower the percentage of alpha crystallites, the better the optical properties of the final parts. For equal degrees of crystallinity, worse optical properties occur for the higher crystallite dimensions [15]. Another strategy consists of varying the injection temperature and mold temperature in order to affect the cooling rate [15,22,25]. Several recent studies on the crystallization kinetics of semi-crystalline thermoplastic materials concluded that very low cooling rates promote the growth of crystalline zones, allowing the spherulites to reach significant dimensions. On the other hand, high cooling rates inhibit their growth, leading to the development of many small spherulites [15,18,23,25,26,27]. In terms of crystalline structure, it has been demonstrated that processing at high shear rates promotes the appearance of the beta form; however, because in conventional processes it is not possible to process a material at high shear rates throughout the entire thickness of the part, this route is not an option [22]. Another way to promote the appearance of beta type spherulites is to slowly cool down the part near 120 °C; this is not feasible at the industrial level since high production rates are required [23]. The most feasible way to promote the beta form is through the incorporation of specific additives. In practical terms, materials with large amounts of beta phase have a lower stiffness, higher yield and rupture deformation, higher impact resistance and better optical properties (higher transparency) [15,24].

Polypropylene (PP) is one of the most widely used thermoplastic materials in the world, accounting for approximately 20% of total plastic material consumption [2]. This material is extremely versatile and inexpensive. It is a semi-crystalline polymer of the polyolefin family with low chemical reactivity, making it suitable for use in contact with strong acids and bases (even at high temperatures). This material is also an excellent candidate for food contact and medical devices. It is simple to color and to process with good thermal stability and low density [7,8,28]. As for the optical and mechanical properties of PP, they vary according to its nature (homopolymer, hPP, and copolymer, rPP) and according to the chemical structure of the polymer chains and polymerization conditions. Thus, PP can adopt three types of spatial configurations (tacticity) of the repetitive units: isotactic, syndiotactic and atactic [7,8,10,29]. This structure is related to the arrangement of the methyl group along the polymer chain, and the presence/absence of other monomers in the main chain, besides propene [28]. As a result, PP can be transparent, opaque, rigid or flexible, which makes it the ideal candidate for the present study [7,19,28,30].

In this work, several commercial PP grades were pre-selected in order to find the one that allows us to obtain greater ranges of haze, or turbidity, a property inversely related to transparency. The variation of turbidity in the injection-molded samples will be induced through the use of different cooling rates/thermomechanical environments since they are expected to result in different degrees of crystallinity. This will be later explored in a special mold enabling us to set/control different simultaneous local temperatures in the same cavity. The innovative character of this work is, therefore, the systematic research, performed with PP grades, with the objective of obtaining different degrees of transparency in the same injected component.

If the primary goal of this research is met, several advantages will result for the part design stage. In fact, the use of a single material with property gradient eliminates the existence of interfacing zones; also, the process and mold will be simpler since bi-injection will not be required. Additionally, it will facilitate the selection of materials, avoiding the need to select compatible or incompatible pairs of materials, according to the purpose of the part. Finally, it will also contribute to a circular economy and facilitate the recycling of components at the end of the product’s useful life.

Potential applications of this research include opaque covers with a local transparent zone that allows for the viewing of an internal digital display, or opaque containers for liquids with a transparent strip that allows for the checking of the level of liquid, among others. 

## 2. Materials and Methods

### 2.1. Materials

The polypropylene grades used in this study are listed in Table 1. All of the grades are commercially available and suitable for injection molding, the technology envisaged for future applications. These grades were chosen to have different material types (homopolymers and copolymers) and a wide range of melt flow indexes (MFI).

### 2.2. Part Geometry and Injection Molding

**Figure 1:**
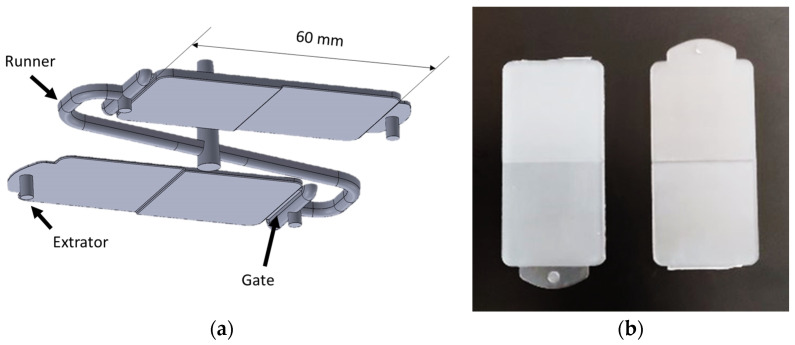
Injection molding of the samples: (**a**) representation of the complete molding; (**b**) examples of injected plastic samples.

The injected part is a 60 mm long rectangle made up of two squares with 30 mm sides, designed for direct use in the equipment available for the evaluation of transmissivity. The mold consists of two interchangeable plates, with two cavity samples on each plate (see Figure 1a), and two different thicknesses on each injected sample, visible in Figure 1b. In one of the plates, one of the parts has 0.5 and 1.0 mm thicknesses, and the other part is 1.5 and 2.0 mm thickness. In the other plate the thicknesses of the two parts is 2.5 and 3.0 mm, and 3.5 and 4.0 mm. Therefore, this mold makes it possible to produce parts with thicknesses ranging from 0.5 mm to 4.0 mm, at 0.5 mm intervals, in order to investigate the effect of this parameter on transmissivity. The parts, illustrated in Figure 1b, were produced in an injection molding machine BOY, Berlin, Germany, model 22 A, with a clamping force of 22 tons.

Each material grade (Section 2.1) was injected with four different processing conditions by varying the injection temperature (Tinj) and the mold temperature (Tmold). The selected temperatures were 210 °C and 250 °C for Tinj, and 25 °C and 90 °C for Tmold, combined in four different processing conditions. For each material, the injection temperatures used are the materials’ minimum and maximum limits, according to the datasheet information, in order to achieve the greatest possible variation of transparency. Table 2 shows the remaining reference processing conditions used. Considering the number of different processing conditions (four) and thicknesses (eight), 32 different types of samples were produced with each of the seven PP grades.

The moldings were manually removed from the mold after the cooling stage, and their temperatures were monitored and recorded using a FLIR SC640 thermographic camera (FLIR, Hudson, NH, USA) throughout the subsequent cooling process. Frames were taken every 10s, allowing us to characterize the actual cooling process undergone after extraction. Measurements were taken on at least three samples of each condition, and the results were averaged.

### 2.3. Samples Characterization

#### 2.3.1. Optical Properties

For the characterization of the injected samples transparency, haze has been considered. Haze is commonly referred to as the amount of visible light that is scattered at angles greater than 2.5° (2.5° < θ < 90°). A haze meter XL-211 Hazegard System, produced by BYK Gardner (BYK-Gardner, Geretsried, Germany), was used following the ASTM D1003 standard [31]. This device records values of the total transmission of light passing through the sample as well as the amount of light that is scattered. To obtain the final haze value, the errors associated with the measurement process were corrected and then the diffuse transmittance value was divided by the total transmittance value. For each condition, at least five samples were tested and the average value was calculated. Other optical properties, such as total, direct and diffuse transmittance, were compared. However, it was concluded that haze/turbidity is the one that best translates the set of values obtained into the real perception of the resulting transparency of the injected samples since it also takes into account the light scattered.

#### 2.3.2. Morphology

**Figure 2:**
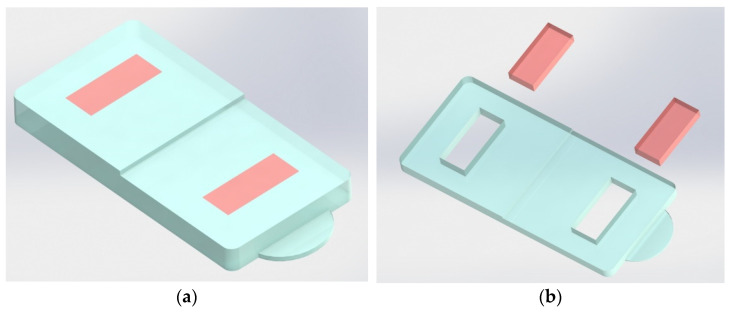
Injected parts and samples (orange blocks) collection for DSC: (**a**) injected part and location of the DSC samples; (**b**) part after the samples’ removal.

Differential scanning calorimetry (DSC) was used to provide information about the degree of crystallinity of the injected samples. Only a first scan was performed, in order to preserve and assess the effect of the thermomechanical conditions in which processing occurred. For this purpose, a DSC 200 F3 Maia, from NETZSCH (Erich NETZSCH GmbH & Co, Selb, Germany) was used, heating the samples from 30 °C to 200 °C at a heating rate of 10 K/min, which is enough of a range to cover where the total melting of the crystalline zones occurs. Figure 2 shows the part regions where the samples were taken.

The raw materials, as pellets, were also characterized under two different conditions: (i) heating at 10 K/min followed by cooling at 10 K/min; and (ii) heating at 10 K/min followed by cooling at 40 K/min using, at least, three samples for each condition. In this case, the first heating was used to eliminating the thermal history of the pellets.

Polarized light microscopy was used to characterize the internal structure of the injected samples in terms of the oriented shell/core ratio, as well as to measure the size of the spherulites. The samples were prepared using a microtome and fixed on a slide with the help of Canada balsam. Subsequently, these samples were observed in a transmission optical microscope LEICA DM 2500P (Leica-microsystems, Wetzlar, Hessen 35578, Germany) coupled to a polarizer. Images were taken using Leica Application Suite software (Leica-microsystems, Wetzlar, Hessen 35578, Germany) with magnification objectives of 2.5×, 4×, 10× and 20×, and an eyepiece of 1.63×. 

These tests, DSC and polarized light microscopy, were only performed on the samples that showed the maximum absolute haze values injected with the PP070G2M grade, and also on the samples produced with the material that originated the wider range of haze, DR 7037.

## 3. Results and Discussion

### 3.1. Materials Screening

**Figure 3:**
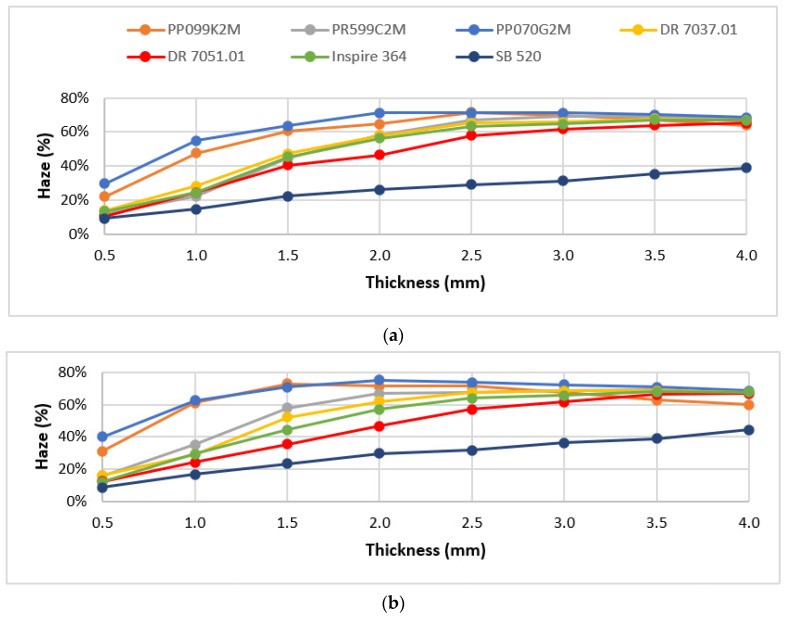

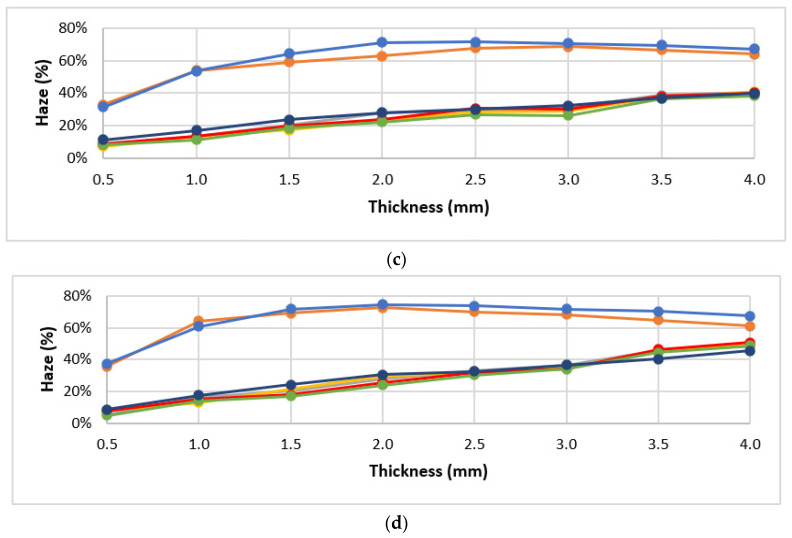
Haze as a function of samples thickness, for the seven PP grades and different processing conditions: (**a**) Tinj = 210 °C, Tmold = 25 °C; (**b**) Tinj = 210 °C, Tmold = 90 °C; (**c**) Tinj = 250 °C, Tmold = 25 °C; (**d**) Tinj = 250 °C, Tmold = 90 °C.

Figure 3 shows the haze values obtained for all tested materials, identified by different colors, and for all processing conditions (Tinj and Tmold combinations). Each point represents the experimental mean value. The standard deviation of the mean values is not shown because it is very low in the majority of cases (0.04–0.20%), reaching a maximum value of 2% for only two of the conditions.

As can be seen, for lower injection temperatures (210 °C, Figure 3a,b), a greater haze variation is observed among all of the materials. Additionally, a wide range of haze is obtained when only the material grade variable is considered, especially for lower thicknesses (from 0.5 mm to 2.5 mm). For the remaining thicknesses, the different material grades exhibited similar haze values, with the exception of grade SB 520. As for the parts injected at 250 °C, Figure 3c,d, there are two distinct behaviors regarding the haze values obtained, which vary according to the nature of the polypropylene, i.e., PP homopolymer and PP random copolymer. The light blue (PP070G2M) and orange (PP099K2M) curves represent the homopolymer grades, whose haze values were the highest for all the processing conditions. The grade that shows the lowest haze is the SB 520. However, the haze property of this grade is not very sensitive to the processing conditions, which does not make it a suitable candidate for the next phase of this work. The material grade with the highest absolute haze is the PP070G2M, which for typical injection molding thicknesses (from 0.5 mm to 1.5 mm), achieves absolute variations in the order of 10%, which, although small, are still interesting for this study. Table 3 shows the grade that presents the wider range of haze variation, as well as the corresponding processing conditions, for samples with a thickness of 3 mm. Note that this 39.5% absolute variation of the property corresponds to a relative variation of more than 100%. 

According to the results obtained, the most promising polypropylene grades were selected for the next phase of the project. These were the DR 7037.01, the material with the widest range of haze variation, and the PP070G2M, which has a different nature from the first, has the highest absolute value of haze among all the materials tested (75.3% at Tinj 210 °C and Tmold 90 °C), and shows one of the widest ranges of haze for the lowest thickness (0.5 mm).

### 3.2. Optical Performance in Use

**Figure 4:**
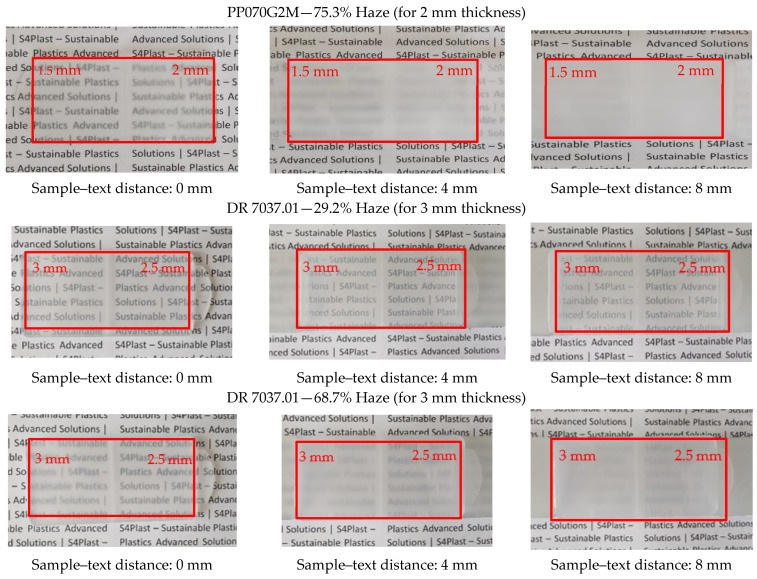
Selected PP grades: effect of the haze value on the optical performance of injected specimens, illustrated for various sample–text distances. Note: each specimen has two distinct thicknesses (see Section 2.2).

The injected samples were placed at various distances in front of a printed text to determine the effect of different haze values on their optical performance. Photographs of these experiments were taken and are shown in Figure 4. The left column, center column and right column correspond to sample–text distances of 0 mm, 4 mm and 8 mm, respectively. In the first line, the sample with thicknesses of 1.5 and 2 mm (as identified) was used, and in the remaining lines, specimens with thicknesses of 2.5 and 3 mm were used.

The effect of the thickness, and especially of the distance between the specimen and the text, is evident. These parameters have a significant impact on the clarity of the observation, which can play a key role in future applications. The differences in haze obtained with the different selected PP grades and with the different processing conditions are also remarkable.

**Figure 5:**
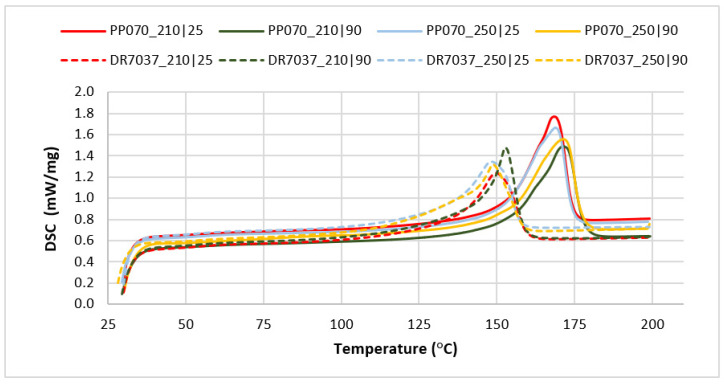
DSC curves obtained in this study (average values taken from the first scan of the injected samples).

To investigate the causes of the haze variation, DSC tests and optical microscopy were used to characterize the morphology of the specimens for the selected grades. The results obtained using DSC are shown in Figure 5 and Table 4.

The curves presented in Figure 5 form two groups, each being representative of one of the two grades selected for further characterization. The first group, with the exothermic peak appearing at a lower temperature, corresponds to the copolymer polypropylene (rPP, DR 7037.01); the second one comprises the curves of the homopolymer polypropylene (hPP, PP070G2M). This difference, occurring in materials with the same chemical structure but with different nature, was expected and is due to the presence of a well-developed crystalline phase in hPP (material characterized by a more regular chain structure), which requires a greater amount of energy to melt.

Analyzing the graphs shown in Figure 5, it can be highlighted that for a given grade, and regardless of the processing conditions, there are no major variation both in the melting temperature and the melting enthalpy (excluding the sample injected at 250 °C with a mold temperature of 90 °C). On the other hand, the homopolymer (PP070G2M) has a higher crystallinity than the copolymer grade (DR 7037.01), which was expected due to their different inherent propensity to crystallize. In any case, the differences in crystallinity, while significant, do not appear to be sufficient to justify the haze differences observed. DSC of the raw materials (granules) was also performed to better investigate this issue. Table 5 shows these results.

The first heating is intended to clean the thermal history of the materials. The second and third heating cycles refer to heating at 10 K/min, after cooling at 10 K/min and 40 K/min, respectively. These results are comparable to those obtained from the injected samples. The observed differences can be attributed to the higher cooling rates occurring in injection molding. This supports the hypothesis that haze differences are determined by factors other than the crystallization degree. 

**Figure 6:**
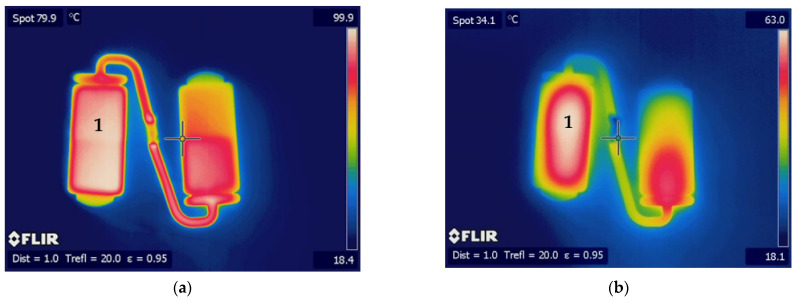

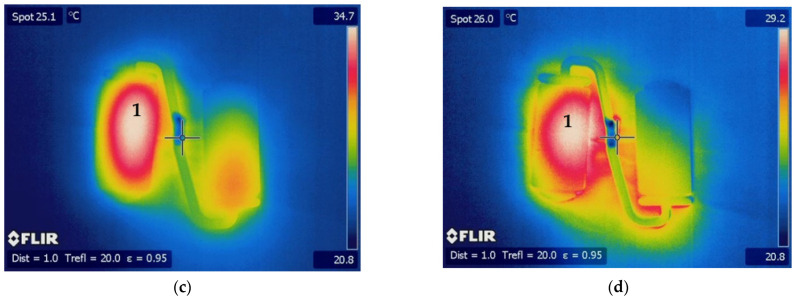
Thermograms corresponding to moldings injected with DR 7037.01 at 210 °C in a mold at 90 °C: (**a**) immediately after demolding; (**b**) 2 min after demolding; (**c**) 4 min after demolding; (**d**) after reaching a maximum temperature of 30 °C.

**Figure 7:**
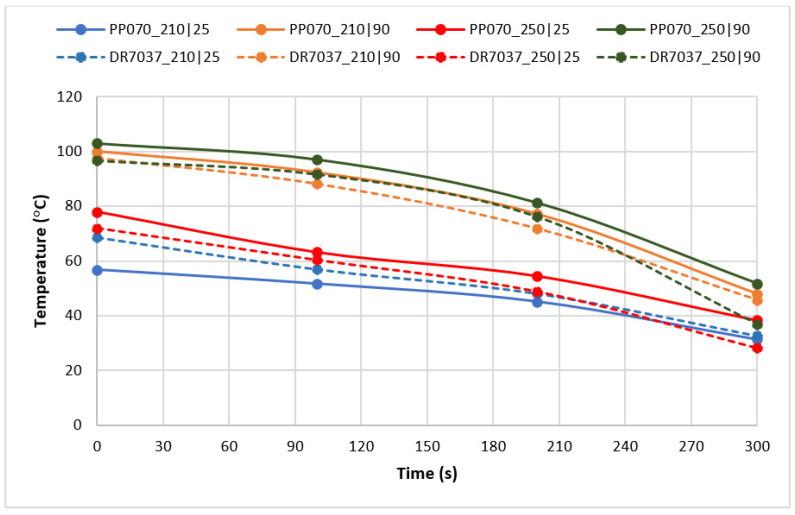
Part temperature variation with time, after demolding, at location “1” (identified in Figure 6).

In order to further investigate the causes of haze differences, the uncontrolled cooling that occurs after the parts’ demolding was monitored with the help of a thermographic camera. Figure 6 shows a typical sequence of the images taken, and Figure 7 shows the evolution of temperature at location “1”, corresponding to the thicker region of the sample, as a function of time.

Considering the left specimen, where the highest turbidity was registered, it can be observed that the values of temperature and its distribution varies along the cooling time. After demolding, the temperature of the part is almost homogeneous. After two minutes of demolding, the highest temperature zone is located in the central zone of the part, since natural convection cools down the external contour more efficiently. At the end of the monitored cooling time, and for the same reason, the maximum temperature continues to occur in the center of the parts. This behavior is common to all samples tested.

As shown in Figure 7, it takes more than five minutes for the temperature to drop to room temperature after demolding. Thus, cooling after demolding may be more important in terms of part crystallinity/turbidity than cooling that occurs in a controlled manner inside the mold.

**Figure 8:**
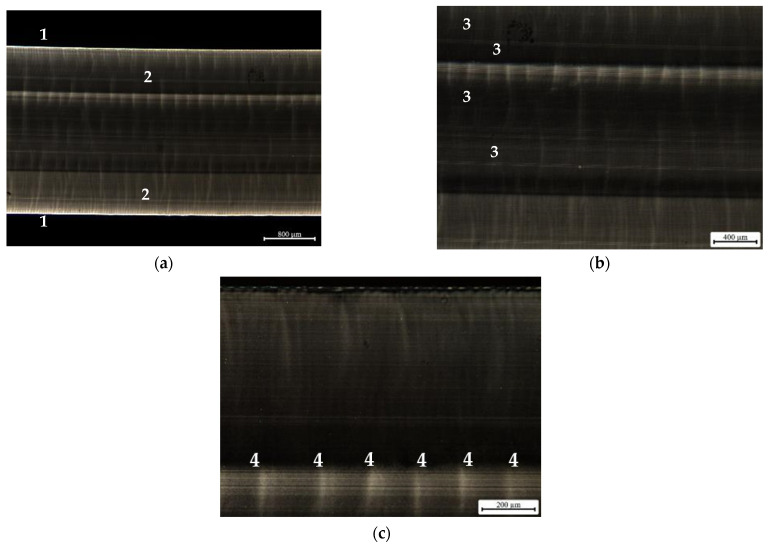
Polarized light microscopy images corresponding to the 3 mm thickness copolymer grade DR 7037.01 specimen, injected at Tinj = 250 °C and Tmold = 25 °C, observed at various magnifications: (**a**) complete sample; (**b**) magnification of the center region of the sample; (**c**) magnification of the bottom region of the sample. Notes: 1—oriented sample shell; 2—sample under-shell; 3—different bands observed; 4—marks promoted by the microtome.

**Figure 9:**
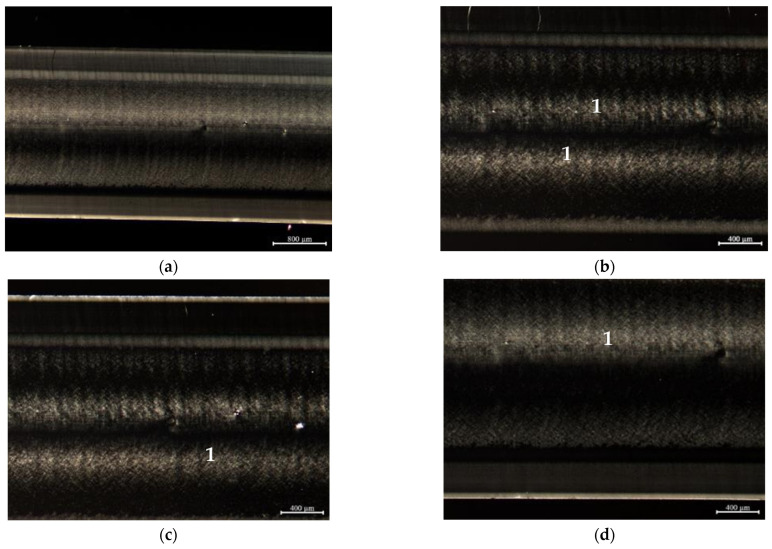
Polarized light microscopy images corresponding to the 3 mm thickness copolymer grade DR7037.01 specimen, injected at Tinj = 210 °C and Tmold = 90 °C: (**a**) complete sample; (**b**) magnification of the center region of the sample; (**c**) magnification of the upper region of the sample; (**d**) magnification of the bottom region of the sample. Note: number 1 identifies zones of well-developed spherulites.

**Figure 10:**
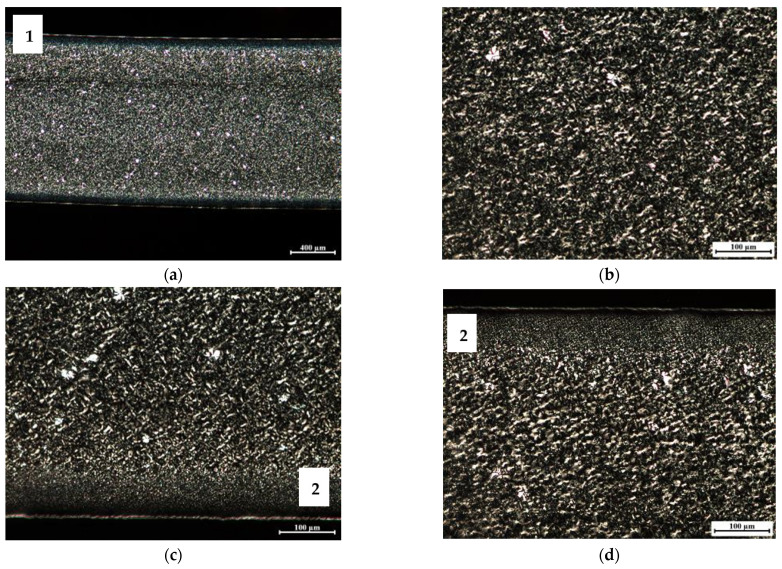
Polarized light microscopy images corresponding to the 2 mm thickness homopolymer grade PP070G2M specimen, injected at Tinj = 210 °C and Tmold = 90 °C: (**a**) complete sample; (**b**) magnification of the center region of the sample; (**c**) magnification of the bottom region of the sample; (**d**) magnification of the upper region of the sample. Notes: 1—oriented sample shell; 2—zone of well-developed spherulites.

In order to assess the morphological changes induced in the specimens (by processing and by the cooling in the environment), polarized light optical microscopy was used. This analysis intends to characterize the specimens’ internal structure, in terms of the oriented shell/core ratio, as well as to check if there is any apparent variation in the size of the spherulites developed. The obtained micrographs are shown in Figure 8, Figure 9 and Figure 10.

The presence of crystalline zones, evidenced by bright points, is not observed in the images shown in Figure 8. However, several bands along the cross section of the sample, identified with ‘3′, can be seen in image (b). Image (a) clearly shows the presence of a very oriented and thin shell, ‘1′, and an under-shell on both sides of the sample, ‘2′. The small marks visible along the width of the sample, visible in image (c), ‘4′, were promoted by sectioning in the microtome during sample preparation. These images demonstrate what was expected for the polypropylene copolymer, that is, the lack of well-developed crystallites.

The micrographs shown in Figure 9, corresponding to the same material injected at a lower temperature (Tinj = 210 °C) in a mold at a higher temperature (Tmold = 90 °C), are those that better evidence distinct layers formed along the cross-section of the samples during cooling. One possible explanation for the formation of these layers is the successive reheating of the already solidified zones by the hotter inner zones during cooling outside the mold. This hypothesis was confirmed by measurements performed with the thermographic camera. For these processing conditions, there are also prominent white spots in the core of the samples, identified with ‘1′, denoting the existence of well-developed spherulites. This is a consequence of the corresponding lower cooling rate that resulted from a high mold temperature.

The micrographs corresponding to the homopolymer PP070G2M are depicted in Figure 10. These reveal well-developed spherulites throughout all of the sample’s cross-section. The presence of a very thin and oriented shell, ’1′, as well as a transition zone between this shell and the core of well-developed spherulites, ‘2′, comprising a large number of smaller sized spherulites, are also noticeable.

The differences in the degree of crystallinity between the two PP grades observed in the micrographs are essentially apparent since they are not supported by the DSC results, presented in Table 4. Additionally, the considerable differences obtained for the haze (of the order of 39.5%) cannot be only attributed to differences in the degree of crystallinity. In fact, these differences may also be promoted by the layered zones, as revealed by the micrographs. These layers are expected to have different refractive indexes and, therefore, they deviate the light propagation during its passage through the injected specimens [15]. Furthermore, the higher the number of layers developed, the more times light is refracted, contributing to the increase in haze. Because it promotes light diffusion and reflection, the interface between layers can also contribute to an increase in haze.

## 4. Conclusions

This work assessed the potential of polypropylene injection molding grades to produce parts with varied transparency (haze) in order to obtain, in the future, mono-material products with a property gradient. Several polypropylene grades of different types were identified and the haze of parts injected under various thermal processing conditions was characterized. The results showed a significant dependence of haze on the tested materials, thermal processing conditions and thicknesses of the samples. The optical performance of the parts when used at various distances from a text (0, 4 and 8 mm) also revealed great differences. This feature has an interesting potential for future applications.

There were no significant differences in the degree of crystallinity measured via DSC capable to justify the differences observed in haze. However, microscopy results revealed the presence of several layers across the samples thickness, for copolymer grade DR 7037.01, or the presence of large spherulites, for homopolymer grade PP070G2M, that determined the samples’ haze. These results should be related to the cooling rate of the parts, after demolding, which, under the studied conditions, takes about 5 min to reach a temperature of 30 °C.

The promising results obtained confirm the potential of using one single material to obtain parts with an optical property (haze) gradient. However, to accomplish this objective further developments and studies will be required. A mold with independent controlled temperatures in different cavity zones is already being designed for this purpose.

## Figures and Tables

**Table 1:** Polypropylene grades used in the study.

Material Reference	Producer	Type	MFI (g/10 min) @230 °C, 2.16 kg
Isplen PP070G2M	Repsol	Homopolymer	12
Isplen PP099K2M	Repsol	Homopolymer	55
Isplen PR599C2M	Repsol	Random Copolymer	75
DR 7037.01	Braskem	Random Copolymer	23
DR 7051.01	Braskem	Random Copolymer	10
Inspire 364	Braskem	Random Copolymer	42
SB 520	Lotte Chemical	Random Copolymer	1.8

**Table 2:** Fixed processing conditions used in the injection molding of samples.

Sample Thickness (mm)	Injection Speed (mm/s)	Packing Pressure (MPa)	Packing Time (s)	Cooling Time (s)
0.5–2.0	80	25	6	22
2.5–4.0	65	20	6	22

**Table 3:** Absolute haze ranges for the grade with wider range of haze variation (DR 7037.01) with a 3 mm thickness.

Processing Conditions	Minimum Haze (%)	Maximum Haze (%)	Haze Range (%)
Value	29.2	68.7	39.5
Tinj|Tmold (°C)	250|25	210|90	

**Table 4:** Thermal properties and degree of crystallization of the PP specimens. Note: this property was computed as the ratio of the specimen melting enthalpy and the melting enthalpy of 100% crystallized PP, i.e., 207 J/kg [32,33,34].

Material	Thickness (mm)	Injection Temperature (°C)	Mold Temperature (°C)	Melting Enthalpy (J/g)	Melting Temperature (°C)	Degree of Crystallization (%)
PP070G2M	2	210	25	90.01 ± 0.03	168.3 ± 0.09	43.48 ± 0.02
PP070G2M	2	210	90	94.82 ± 0.04	171.8 ± 0.15	45.80 ± 0.01
PP070G2M	2	250	25	87.77 ± 0.03	168.9 ± 0.02	42.40 ± 0.002
PP070G2M	2	250	90	102.25 ± 0.03	161.3 ± 0.34	49.40 ± 0.01
DR 7037.01	3	210	25	81.25 ± 0.06	150.34 ± 0.21	39.25 ± 0.04
DR 7037.01	3	210	90	90.78 ± 0.01	152.8 ± 0.08	43.86 ± 0.002
DR 7037.01	3	250	25	83.66 ± 0.11	148.3 ± 0.10	40.41 ± 0.06
DR 7037.02	3	250	90	85.51 ± 0.02	150.4 ± 1.73	41.31 ± 0.01

**Table 5:** Dependence of the thermal properties and degree of crystallization on the heating cycle of the raw materials (granules). Note: this property was computed as the ratio of the specimen melting enthalpy and the melting enthalpy of 100% crystallized PP, i.e., 207 J/kg [32,33,34].

Material	Heating Cycle	Melting Enthalpy (J/g)	Melting Temperature (°C)	Degree of Crystallization (%)
PP070G2M	1st	84.825 ± 0.01	168.9 ± 0.4	40.98 ± 0.02
PP070G2M	2nd	95.17 ± 0.02	168 ± 0.004	45.98 ± 0.01
PP070G2M	3rd	89.405 ± 0.02	168.4 ± 0.36	43.19 ± 0.01
DR 7037.01	1st	91.02 ± 0.01	152.8 ± 0.14	43.97 ± 0.04
DR 7037.01	2nd	87.015 ± 0.01	151.2 ± 0.02	42.04 ± 0.05
DR 7037.01	3rd	79.995 ± 0.01	149.8 ± 0.25	38.64 ± 0.05

## References

[1] Böll H. (2019). Plastic Atlas 2019: Facts and Figures about the World of Synthetic Polymers.

[2] Plasctics Europe (2021). Plastics—The Facts 2021: An Analysis of European Plastics Production, Demand and Waste Data.

[3] Carrington D. After Bronze and Iron, Welcome to the Plastic Age, Say Scientists. The Guardian. 4 September 2019. https://www.theguardian.com/environment/2019/sep/04/plastic-pollution-fossil-record.

[4] Culter J.D., Selke S.E.M. (2016). Plastics Packaging Book.

[5] Thompson R.C., Swan S.H., Moore C.J., Vom Saal F.S. (2009). Our plastic age. Philos. Trans. R. Soc. B Biol. Sci..

[6] Wypych G. (2016). Handbook of Polymers.

[7] Maier C., Calafut T. (1998). Polypropylene The Defenitive User’s Guide and Databook.

[8] Osswald T.A., Menges G. (2012). Material Science of Polymers for Engineers.

[9] Encyclopaedia Britannica (2020). The Composition, Structure, and Properties of Plastics.

[10] Harper C.A. (2002). Handbook of Plastics, Elastomers, and Composites.

[11] Bakas I., Ferna R., Reichel A., Trier X., Zeiger B. (2021). Plastics, the Circular Economy and Europe’s Environment—A Priority for Action.

[12] Pattanaik S.S., Sangavi S. (2020). Marine habitat destruction: An anthropogenic way towards the end of life in the ocean. Vigyan Varta.

[13] Sheavly S.B., Register K.M. (2007). Marine debris & plastics: Environmental concerns, sources, impacts and solutions. J. Polym. Environ..

[14] Korniejenko K., Kozub B., Bak A., Balamurugan P., Uthayakumar M., Furtos G. (2011). Tackling the Circular Economy Challenges-Composites Recycling: Used Tires, Wind Turbine Blades, and Solar Panels. J. Compos. Sci..

[15] De Santis F., Pantani R. (2013). Optical properties of polypropylene upon recycling. Sci. World J..

[16] Molnár J., Sepsi Ö., Erdei G., Lenk S., Ujhelyi F., Menyhárd A. (2020). Modeling of light scattering and haze in semicrystalline polymers. J. Polym. Sci..

[17] Lin Y., Bilotti E., Bastiaansen C.W.M., Peijs T. (2020). Transparent semi-crystalline polymeric materials and their nanocomposites: A review. Polym. Eng. Sci..

[18] Gahleitner M., Grein C., Kheirandish S., Wolfschwenger J. (2011). Nucleation of Polypropylene Homo-and Copolymers. Int. Polym. Process..

[19] Luo S., Wei L., Sun J., Huang A., Qin S., Luo H., Gao C., Zheng Y., Shen J. (2019). Crystallization behavior and optical properties of isotactic polypropylene filled with α-nucleating agents of multilayered distribution. RSC Adv..

[20] Zia Q., Androsch R., Radusch H.J. (2010). Effect of the structure at the micrometer and nanometer scales on the light transmission of isotactic polypropylene. J. Appl. Polym. Sci..

[21] Menyhárd Z.H.A., Doshev P., Gahleitner M., Vörös G., Varga J., Pukánszky B. (2014). Effect of the molecular structure of the polymer and nucleation on the optical properties of polypropylene homo-and copolymers. ACS Appl. Mater. Interfaces.

[22] Tordjeman P., Robert C., Marin G., Gerard P. (2001). The effect of alpha, beta crystalline structure on the mechanical properties of polypropylene. Eur. Phys. J. E.

[23] Gradys A., Sajkiewicz P., Minakov A., Adamovsky S., Schick C., Hashimoto T., Saijo K. (2005). Crystallization of polypropylene at various cooling rates. Mater. Sci. Eng. A.

[24] Papagiorgiou D.G., Chrissafis K., Bikiaris D. (2011). β-Nucleated Polypropylene: Processing, Properties and Nanocomposites. Polym. Rev..

[25] Lamberti G. (2011). Isotactic polypropylene crystallization: Analysis and modeling. Eur. Polym. J..

[26] Nielsen A.S., Pyrz R. (1998). The effect of cooling rate on thermal residual strains in carbon/polypropylene microcomposites. Sci. Eng. Compos. Mater..

[27] Brucato V., Piccarolo S., La Carrubba V. (2002). An experimental methodology to study polymer crystallization under processing conditions. The influence of high cooling rates. Chem. Eng. Sci..

[28] Tripathi D. (2002). Practical Guide to Polypropylene.

[29] Maddah H.A. (2016). Polypropylene as a Promising Plastic: A Review. Am. J. Polym. Sci..

[30] Ahmed A.K., Atiqullah M., Pradhan D.R., Al-Harthi M.A. (2017). Crystallization and melting behavior of i-PP: A perspective from Flory’s thermodynamic equilibrium theory and DSC experiment. RSC Adv..

[31] (2021). Standard Test Method for Haze and Luminous Transmittance of Transparent Plastics.

[32] van der Meer D.W. (2003). Structure-Property Relationships in Isotactic Polypropylene. Ph.D. Thesis.

[33] Ehrenstein G.W., Theriault R.P. (2001). Polymeric Materials: Structure, Properties, Applications.

[34] Ruiz-Orta C., Fernandez-Blazquez J.P., Anderson-Wile A.M., Coates G.W., Alamo R.G. (2011). Isotactic polypropylene with (3,1) chain-walking defects: Characterization, crystallization, and melting behaviors. Macromolecules.

